# Diversity of Catechin in Northeast Indian Tea Cultivars

**DOI:** 10.1100/2012/485193

**Published:** 2012-02-14

**Authors:** Santanu Sabhapondit, Tanmoy Karak, Lakshi Prasad Bhuyan, Bhabesh Chandra Goswami, Mridul Hazarika

**Affiliations:** ^1^Department of Biochemistry, Tocklai Experimental Station, Tea Research Association, Assam, Jorhat 785008, India; ^2^Department of Soil, Tocklai Experimental Station, Tea Research Association, Assam, Jorhat 785008, India; ^3^Department of Chemistry, Gauhati University, Assam, Guwahati 781014, India

## Abstract

Tea (*Camellia sinensis* L.) leaf contains a large amount of catechins (a group of very active flavonoids) which contribute to major quality attributes of black tea. Based on morphological characters tea plants were classified as Assam, China, and Cambod varieties. The present study is an attempt for biochemical fingerprinting of the tea varieties based on catechin composition in green leaf of cultivars grown in Northeast India. Assam variety cultivars contained the highest level of catechins followed by Cambod and China. The average catechin contents were 231 ± 7 mg g^−1^, 202 ± 5 mg g^−1^, and 157 ± 4 mg g^−1^ of dry weight of green leaf for Assam, Cambod, and China cultivars, respectively. Among the individual catechins the variations in epigallocatechin gallate (EGCG) and epigallocatechin (EGC) were the most prominent among the varieties. High EGC content was found to be a characteristic of Assam variety which was further corroborated through multivariate analysis.

## 1. Introduction

Present market is a selective one, and producers with high-quality tea are likely to survive. Quality of made tea of the plains of Northeast (NE) India depends on the quality of raw materials determined primarily by the polyphenolic constituents. It is widely accepted that Crush, Tear, and Curl (CTC) black tea quality attributes depend on flavonol composition (more precisely catechins). Epigallocatechin gallate (EGCG) is an important biochemical marker of Northeast Indian tea as it contributes 50% of total catechins [1]. The state of Assam (26°4′N–27°30′N and 89°58′E–95°41′E) in India is one of the major tea producing areas of the world. Tea in NE India is processed largely from the leaf of *Camellia assamica*, a source of a wide range of the catechins. Tea leaves contain about 180–360 mg g^−1^ of polyphenols, among which 70–80% are flavanols [2]. Total polyphenols including composition of catechins as well as their oxidation products were identified as being related to tea quality [3–7]. The variation in catechin composition is reflected in the variation in theaflavins (TFs) composition of black tea. It is well established that the formation of dihydroquercetin and dihydromyricetin, which are the precursors of dihydroxy catechins (epicatechin, (EC), epicatechin gallate (ECG)) and trihydroxy catechins (EGC and EGCG), respectively, is under genetic control [8–11]. 

Taxonomically tea is known as *Camellia sinensis* and belongs to the family Theaceae. Commercial tea cultivars are recognized under three different taxa, namely, *C. sinensis*, *C. assamica*, and *C. assamica ssp. lasiocalyx* [12]. However, tea is highly heterogeneous [9], and all the above taxa freely inter-breed, resulting in a cline extending from extreme China types to those of Assam origin [13]. Hybridization has been so extensive that it is often debated if archetypal *C. sinensis*, *C. assamica*, or *C. assamica ssp*.* lasiocalyx* still exist [14]. 

**Figure 1:**
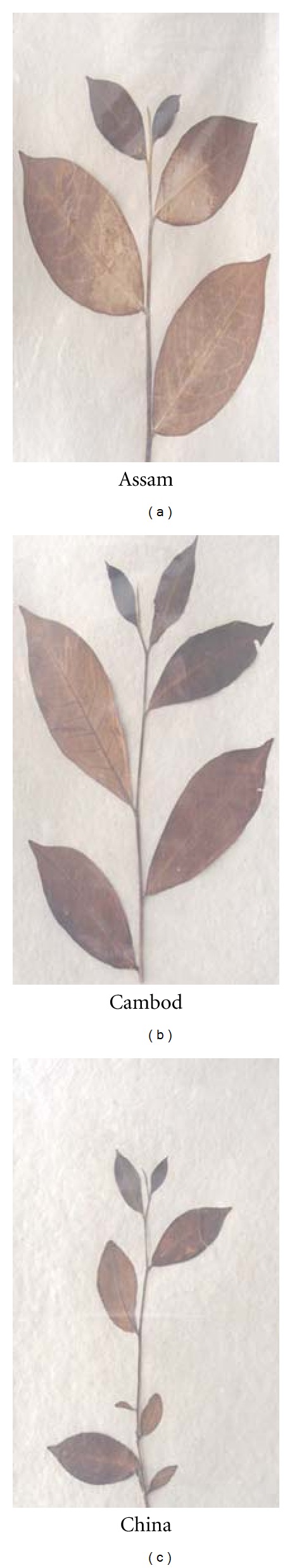
The typical shoots of three varieties (note that the photographs were taken from the herbarium of Tocklai Experimental Station).

Based on morphological characteristics, tea is grouped into Assam, China, and Cambod varieties (Figure 1). The classification has been generally followed in Indian sub-continent possibly because of more varied and heterogeneous tea populations in the region [13]. The genetic differences between the hybrids are well reflected in biochemical composition of leaves. However, biochemical composition as varied between varieties is yet to be fully utilized in tea taxonomy [15]. Presence or absence of certain phenolic substances in the tea shoot has also been used as an aid in establishing interrelationship between taxa [16]. It has been reported that Assam type cultivars contain higher amount of polyphenols [2]. China variety cultivars generally possess quercetin and kaemferol-3-glucosides but these are totally absent or present only in traces in Assam variety [17, 18]. 

**Figure 2:**
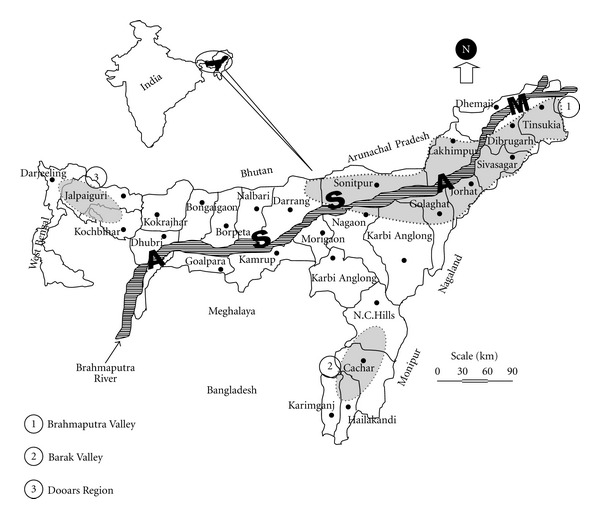
Map showing major tea growing areas of Northeast India.

Tocklai Experimental Station, Jorhat, Assam, has released 153 germplasms to the tea industry of NE India to be grown in plains. Over 60% of 0.3 million ha of tea growing area of NE Indian plains is covered with these tea varieties. Regional variation of quality within the tea growing region (Figure 2) can be attributed to genetic diversity and its interaction with the environment.

Widespread cultivation of clonal tea for high yield and uniform quality may diminish the genetic diversity. Conservation of germplasm resources is necessary for sustainability of the tea industry. Tocklai Experimental Station has a field gene bank with over 2000 germplasms which is one of the primary centres of dispersal in the world. In order to ascertain diversity careful study of secondary metabolites, especially those which are major contributors to quality, is essential. Total catechin content could be used to indicate the quality potential of tea, with high content being related to high quality [4]. Earlier studies showed that tannin content, which is a measure of total catechin contents, could be used in the determination of genetic diversity in tea [19, 20]. However, these methods did not take into account the individual catechins present in tea leaf. Since the formation of black tea quality attributes is influenced by various catechins, characterization of cultivars based on various forms of catechins is essential to identify their quality potential [21]. 

The oxidative and hydrolytic enzymes endogenous to the shoots are crucial in triggering of various characteristic quality attributes of black tea. Out of the various stages of black tea processing, the fermentation stage is the most crucial. The mechanical maceration of green tea shoots triggers the enzyme catalyzed oxidative reactions involving catechins as substrates. Upon disruption of the intercellular compartments, catechins present in the cell vacuole undergo *in vivo* oxidative and hydrolytic processes in presence of mild aeration. The desirable colour and briskness of made tea is dependent on the oxidative polymerization of catechins to TFs and thearubigins (TRs) by the enzymes polyphenol oxidase (PPO) and peroxidase (POD) [22]. 

The present study was undertaken to assess the variation of catechin (viz. EC, ECG, EGC, +C and EGCG) concentrations in extreme and cultivated varieties of Assam, China, and Cambod. The study also took into account the relative expression of individual catechins in cultivars grown in Northeast India. An understanding of catechin profile in different cultivars of tea may provide useful information on plant diversity as well as understanding their role as precursor of quality since type and quantity of catechin significantly influence the formation of two important quality attributes of tea such as theaflavins and thearubigins. This may also support future selection process for improvement of crop quality. 

## 2. Materials and Methods

### 2.1. Plant Materials

Tea shoots comprising apical bud and subtending two leaves were harvested from the experimental garden of Tocklai Experimental Station, Tea Research Association, Jorhat, Assam, India (94°12′E and 26°47′N). A regular 7-day plucking during tea harvesting period was maintained. 7-day plucking interval is a common agricultural practice in tea growing areas of the NE India as it makes the young shoots produce high quality tea [23]. All the sampling plots received identical agricultural practices (fertilization at 120 kg N ha^−1^, 110 kg K ha^−1^ as K_2_O and 30 kg P ha^−1^ as P_2_O_5_ was applied per year, pruning and plucking were maintained) where shade applied over the tea bushes contributed to 30–40% light interception. The reference samples represented pure varieties, namely, Assam (Betjan), China (Vimtal), and Cambod (extreme Cambod). Except reference samples, other cultivars representing the three varieties were as follows.

**Assam Variety**

TV2, TV12, TV13, TV17, TV21, S_3_A_1_, S_3_A_3_, Tingamira, TA 17, and T_3_E_3_ (where TV stands for Tocklai Vegetative and TA stands for Tin Ali).

**China Variety**

TV7, 14/13/3, 14/100/10, 14/100/16, 14/100/6, 317/1, 317/2, 317/3, 317/4, and P126 (P stands for Panitola).

**Cambod Variety**

TV9, TV18, TV22, TV23, TV25, TV26, and TV30.

The entire harvesting period for the sampling was from March to November for the years 2009 and 2010. Leaf samples were collected from the plots receiving similar agronomical practices. Samples were analyzed fortnightly. Soil samples collected from experimental plots were analyzed following the standard procedure described by Jackson [24]. Average soil status of the experimental plots was as follows: well drained sandy loam soil, sand: 57.7 ± 2.1%, silt: 35.5 ± 1.4%, clay: 6.7 ± 0.7%, pH: 4.5 ± 0.002, organic carbon content: 8.0 ± 0.11 mg g^−1^, total nitrogen: 0.8 ± 0.0001 mg g^−1^, available P_2_O_5_: 0.01 ± 0.001 mg g^−1^, and available K_2_O: 0.08 ± 0.0001 mg g^−1^.

### 2.2. Estimation of Catechins

About 100 g fresh tea leaf of each sample was deactivated and dried (at 90°C and dryness around 95%) in a microwave convection domestic oven (Model no. Onida PC21, India). Microwave drying of the samples did not affect catechin composition of green leaf tea samples (unpublished data of Biochemistry Department, Tocklai Experimental Station). The dried samples were ground finely for analysis. 0.2 g of sample was extracted with 5 mL 70% methanol in a water bath at 70°C over 10 min with intermittent shaking in a vortex mixture. The extract was then cooled and centrifuged at 4000 rpm (Rotanta 460R, UK) for 10 min. The supernatant was decanted into a 10 mL volumetric flask. The extraction was repeated twice and volume was made up with the solvent. 1 mL of the extract was diluted to 5 times with stabilizing agent. The stabilizing agent was prepared from EDTA (500 *μ*g mL^−1^), ascorbic acid (500 *μ*g mL^−1^), and acetonitrile (25% v/v) in water. Catechins were quantitatively estimated using waters high-performance liquid chromatography (HPLC) system with Luna 5 *μ* phenylhexyl phenomenax column (4.5 mm × 250 mm) and UV-Vis detector (Waters 2487, USA) set at 278 nm according to the method of International Standard Organisation [25]. During HPLC analysis, 10 *μ*L of the diluted extract was injected into the column through Rheodyne injector. In brief, the elution made was initial 10 min with 100% mobile phase A followed by over 15 min with a linear gradient to 68% mobile phase A and 32% mobile phase B and held at this composition for another 10 min with flow rate 1 mL per min. The mobile phase A consists of 2% acetic acid and 9% acetonitrile and mobile phase B 80% acetonitrile. The chromatographic peaks were identified and estimated by external standard method from response factors (concentration of standards/peak area of standards) determined from different catechin standards procured from Sigma Aldrich, USA (ISO-14502 2005). The solvents used for extraction and analyses were of HPLC grade (E. mark, Mumbai, India).

### 2.3. Statistical Analysis

Raw data of various catechins of analysed tea samples were arranged in a data table where each row referred to an individual, and columns were associated to different variables.

The data were also log-transformed so as to more closely correspond to normal distribution. Further, all the variables were standardized by calculating their standard scores (*z*-scores) as follows:

(1) $$z_{i}=\frac{x_{i}-\overset{®}{x}}{s},$$

where *z*_*i*_ is the standard score of the sample *i*; *x*_*i*_ is the value of sample *i*,  $\overset{®}{x}$ is the mean and *s* is the standard deviation.

Standardization scales the log-transformed data to a range of approximately ±3 standard deviations, centered about a mean of zero. In this way, each variable has equal weight in the statistical analyses. Besides normalizing and reducing outliers, these transformations also tend to homogenize the variance of the distribution [26–28]. Standardization also tends to increase the influence of variables whose variance is small and reduce the influence of variables whose variance is large. Furthermore, the standardization procedure eliminates the influence of different units of measurement and renders the data dimensionless.

The data were used for hierarchical agglomerative cluster analysis (HCA) described by Singh et al. [29 and principal component analysis (PCA) described by Kano et al. [30]. All these statistical analyses were performed using SPSS version 13 (SPSS Inc., Chicago, USA) [31].

## 3. Results and Discussion

Biochemical parameters of green leaf influencing black tea quality of the plains of NE India consist of catechins which are converted to TFs and TRs, the critical parameters of quality of CTC tea [32, 33]. Notwithstanding total polyphenols correlate with black tea quality, some polyphenols do not contribute to the formation of any black tea quality parameter [34]. Only flavan-3-ols are critical for black tea quality [7]. The average catechin compositions of green tea leaves of the cultivars of three varieties is presented in Table 1. Large variations in the catechin compositions were observed among the cultivars reflecting genetic variability [6].

It was observed from this study that the total catechin and some individual catechins could be used as markers to differentiate between the three major varieties. The clear differentiation of China variety from Assam and Cambod could be established using the catechin as marker (see below). Similar observations were reported in Japanese tea [19]. Total green leaf catechin concentration and the ratio of dihydroxy to trihydroxy catechins were used to establish genetic diversity in the tea germplasms of Kenya [6]. Distribution of various catechins in all the three varieties showed that trihydroxy catechins accounted for 71–76% followed by dihydroxy for 22–27%. It is worth mentioning that EGCG which alone contributed 52–58% of total catechins was responsible for higher values of trihydroxy catechins. EGCG accounted for around 55% of total catechins in cultivars grown in Assam which was higher than the Central and Southern African tea leaf and much higher than Kenyan tea where contribution of EGCG was around 25% [5, 7].

Total catechin contents in green leaf were 231 ± 7.40 mg g^−1^, 202 ± 4.58 mg g^−1^, and 157 ± 3.82 mg g^−1^ for Assam, Cambod and China varieties, respectively. Large variations in individual catechins and total catechins among the varieties were observed. Assam variety cultivars contained the highest catechins followed by Cambod, and China (Table 1). The average EGCG contents of the varieties were 121.7 ± 2.4 mg g^−1^ for Assam, 112.6 ± 2.9 mg g^−1^ for Cambod and 86.2 ± 1.3 mg g^−1^ for China. Out of the eleven Assam cultivars studied, EGCG content of the cultivar S_3_A_3_ was found to be the highest. As the results indicated, the catechin content in China variety was substantially lower than the other two varieties.

The second largest contributor to total catechin content was EGC for Assam variety while it was ECG for Cambod and China variety. The variation in EGC content was more prominent between the varieties. The average EGC contents were 51.0 ± 1.0 mg g^−1^ for Assam, 36.1 ± 1.3 mg g^−1^ for Cambod, and 25.7 ± 0.8 mg g^−1^ for China variety. The average ECG content was found lower than EGC in Assam cultivars while in Cambod, and China cultivars it was higher. The average ECG contents in Assam, Cambod and China varieties were 38.6 ± 1.0 mg g^−1^, 37.5 ± 1.2 mg g^−1^, and 30.4 ± 1.2 mg g^−1^ respectively. Therefore, high EGC was a characteristic precursor of Assam variety. The dihydroxy-to-trihydroxy-catechin ratio (CATRAT) among the varieties was between 0.3 and 0.7. The highest CATRAT was found in TV7 of China variety.

### 3.1. Cluster Analysis and Principal Components Analysis

**Figure 3:**
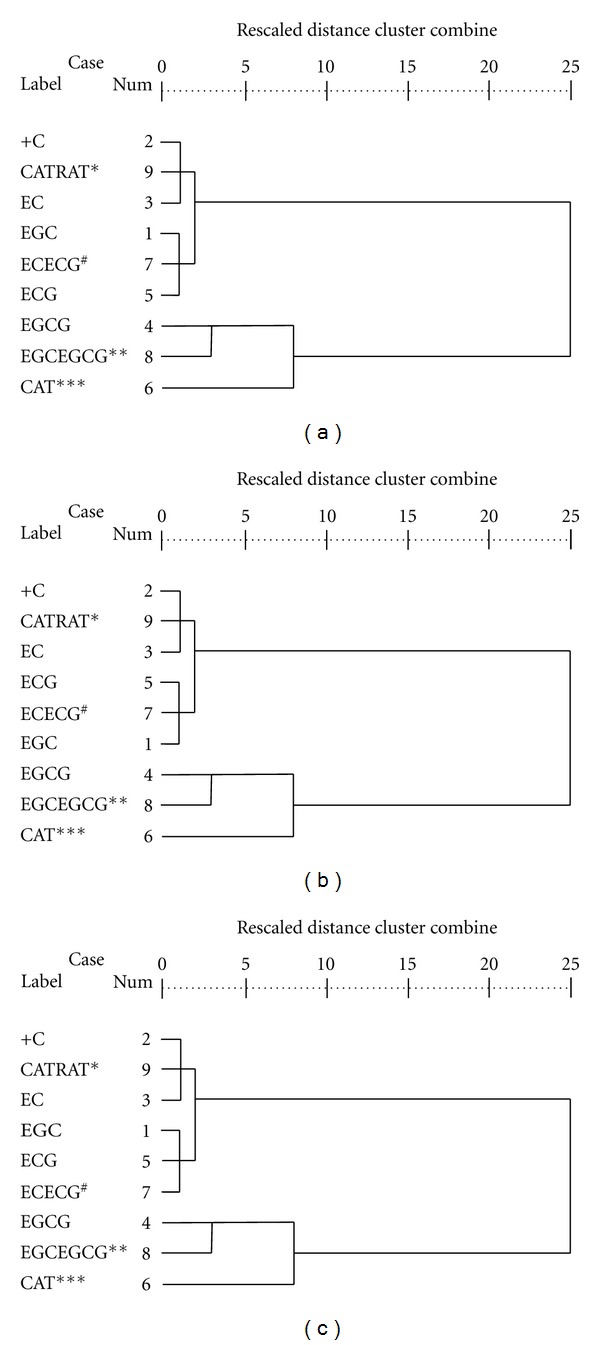
Dendrogram for cluster analysis. The dissimilarity defined by Euclidean distance and the combination of clusters is based on Ward method. (a) Assam, (b) Cambod, and (c) China tea (*CATRAT: catechin ratio; ^#^ECECG: dihydroxy catechin; **EGCEGCG: trihydroxy catechin, and ***CAT: total catechin).

Hierarchical agglomerative cluster analysis (HCA) in the form of dendrogram and principal components analysis (PCA) were used to explore structure and relationships in multivariate data [27, 28]. The rationale of cluster analysis was to partition a set of objects into two or more groups based upon the similarity of the objects in many disciplines with respect to a chosen set of characteristics so that similar objects were in the same class [35]. In the cluster analysis, emphasis was to differentiate biochemical parameters, based upon multiple tea samples and quality parameters, and it was done through HCA. Therefore, HCA was applied to the biochemical data sets with a view to grouping the similar spatial variabilities spread over the variety of tea samples and in the resultant dendrogram. This method used the analysis of variable approach to evaluate the distances between clusters, attempting to minimize the sum of squares of any two clusters that could be formed at each step. It yielded a dendrogram (Figures 3(a), 3(b), and 3(c)), grouping all variables of the samples into two statistically significant clusters. For Assam tea, three clusters were constructed. One cluster included (+) catechin (+C), dihydroxy-to-trihydroxy-catechin ratio (CATRAT) and EC, another one included EGC, dihydroxy catechin (EC + ECG), and ECG. These two clusters were interrelated with another cluster having EGCG, trihydroxy catechin (EGC + EGCG), and total catechin (CAT) (Figure 3(a)). The similar pattern of dendrogram was also observed for Cambod (Figure 3(b)) and China varieties (Figure 3(c)). Therefore, this indicated that all parameters were likely having direct influence on the quality of tea leaf irrespective of their varieties. 

**Figure 4:**
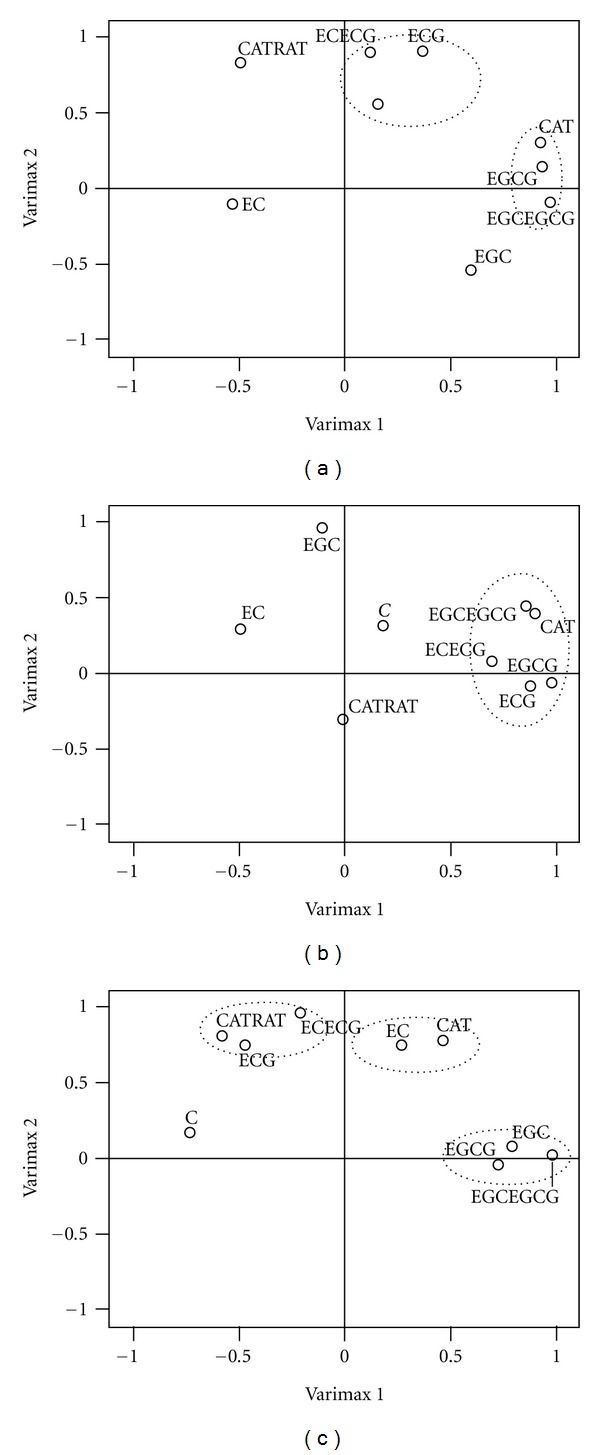
Factor loading pattern of the analyzed variables of the three varieties obtained by PCA followed by Varimax rotation. (a) Assam, (b) Cambod, and (c) China tea (note that in these figures “C” indicates “+C”).

From HCA, we could not clearly distinguish the relations among the different varieties of tea samples. Therefore, all the parameters were transformed into three main comprehensive matrices referring to PCA technique. On plotting the first two principal components (Varimax 1 and Varimax 2), they showed two clusters for Assam tea, one cluster for Cambod tea, and three clusters for China tea in PCA (Figures 4(a), 4(b), and 4(c)). Principal component analysis (PCA) is one of the best statistical techniques for extracting linear relationships among a set of variables. Principal components are the linear combinations of original variables and are the eigenvectors. The Varimax rotation distributes the PC loadings such that their dispersion is minimized by maximizing the number of large and small coefficients. The Cornbach alpha and Kaiser-Meyer-Olkin (KMO) sample adequacy showed the appropriate application of PCA in the present dataset. Principal component 1 (PC1) had higher loadings for the variables like ECG and dihydroxy catechin (EC + ECG) with +C for Assam tea (Figure 4(a)). PC1 accounted for 41.8% of the total variance and could be thus interpreted as a catechin component. PC2 contained 33.6% of the variance and had a higher loading for total catechin (CAT), EGCG, and trihydroxy catechin (EGC + EGCG). This component can be explained taking into account that high levels of total catechin contributed to better quality of Assam tea.  Figure 4(b) reflects the PCA of Cambod tea. Here only one PC (PC1) was obtained containing ECG, EGCG, dihydroxy catechin (EC + ECG), and trihydroxy catechin (EGC + EGCG) with total catechin (CAT). PC1 contained 83% of the variance. Therefore, comparing Figures 4(a) and 4(b) it can be concluded that the pattern of catechins in Cambod tea differed from the one present in Assam tea. China tea gave three principal components. PC1 explained 44.43% of the total variance, whereas PC2 and PC3 expressed 33.37% and 9.70%, respectively, of the variance. PC1 can be interpreted as a major quality component of China tea where the contributing factors were EGC, EGCG, and trihydroxy catechin (EGC + EGCG), as shown in Figure 4(c). 

## 4. Conclusion

Differential display of catechins in cultivars forms a basis for future elucidation of catechin metabolism in tea. Profiling of individual and total catechins was found to be a useful technique to determine genetic diversity in tea germplasms. Among the three pure varieties China variety cultivars contained lower catechins. PCA showed different groupings of catechins for Assam, Cambod, and China teas, and such groupings might be used to differentiate between such varieties.

## Figures and Tables

**Table 1:** Catechin profile of different green tea leaves (all units are in mg g^−1^ except catechin ratio, data represent the mean of three replicates ± standard error).

Cultivar	EGC	+C	EC	EGCG	ECG	Total Catechin (CAT)	Dihydroxy Catechin (EC+ECG)	Trihydroxy Catechin (EGC+EGCG)	CATRAT*
*Assam varieties *									
TV2	53.3 ± 0.5	5.4 ± 0.4	21.2 ± 0.6	114.7 ± 0.9	34.2 ± 0.4	228.1 ± 1.0	55.4 ± 0.5	167.9 ± 1.0	0.3 ± 0.1
TV12	43.2 ± 0.4	6.0 ± 0.2	12.0 ± 0.1	119.8 ± 0.8	36.6 ± 0.8	217.6 ± 0.8	48.6 ± 0.7	163.0 ± 0.5	0.3 ± 0.0
TV13	55.2 ± 0.7	4.5 ± 0.7	12.8 ± 0.3	140.8 ± 1.7	43.0 ± 0.7	256.6 ± 1.5	54.8 ± 0.9	195.9 ± 1.7	0.3 ± 0.0
TV17	41.2 ± 1.6	9.5 ± 0.4	12.5 ± 0.9	129.4 ± 1.1	62.6 ± 1.7	256.0 ± 0.6	75.2 ± 1.6	170.6 ± 1.2	0.5 ± 0.2
TV21	58.3 ± 1.5	4.6 ± 0.5	12.3 ± 0.5	111.2 ± 0.4	34.3 ± 0.3	220.7 ± 0.9	46.7 ± 0.4	169.5 ± 1.1	0.3 ± 0.1
S_3_A_1_	62.3 ± 0.6	3.5 ± 0.2	14.6 ± 0.2	142.5 ± 0.7	46.2 ± 0.9	269.2 ± 0.7	61.3 ± 0.7	212.8 ± 1.5	0.3 ± 0.0
S_3_A_3_	63.1 ± 0.8	7.7 ± 0.1	12.5 ± 0.0	147.0 ± 0.6	43.6 ± 0.9	274.0 ± 0.4	55.4 ± 0.8	210.5 ± 0.2	0.3 ± 0.1
Tingamira	48.4 ± 0.4	5.3 ± 0.5	19.8 ± 0.6	109.7 ± 0.6	35.4 ± 0.3	218.5 ± 0.6	55.2 ± 0.4	158.0 ± 0.5	0.4 ± 0.0
TA17	49.9 ± 0.8	8.6 ± 0.2	10.0 ± 0.1	139.7 ± 0.8	37.9 ± 0.5	246.2 ± 1.0	48.0 ± 0.5	189.8 ± 1.1	0.3 ± 0.0
T_3_E_3_	50.8 ± 1.0	4.1 ± 0.6	20.0 ± 0.6	113.1 ± 0.8	35.0 ± 0.6	222.3 ± 0.9	54.8 ± 0.6	163.9 ± 0.8	0.3 ± 0.1
Betjan	48.4 ± 0.5	4.6 ± 0.4	20.2 ± 0.5	108.0 ± 0.9	34.4 ± 0.7	215.2 ± 0.9	54.6 ± 0.7	156.4 ± 0.9	0.4 ± 0.1
*Cambod varieties *									
TV9	46.6 ± 0.4	4.5 ± 0.8	11.4 ± 0.7	122.6 ± 1.2	35.1 ± 0.7	220.3 ± 1.0	46.5 ± 0.8	169.2 ± 1.1	0.3 ± 0.1
TV18	31.0 ± 0.2	4.3 ± 0.1	5.7 ± 0.1	163.6 ± 0.2	60.8 ± 0.4	265.4 ± 0.3	65.9 ± 0.4	195.5 ± 0.2	0.3 ± 0.0
TV22	46.9 ± 0.1	7.2 ± 0.1	14.1 ± 0.7	131.0 ± 1.0	51.7 ± 0.1	250.9 ± 0.6	65.8 ± 0.2	177.9 ± 0.9	0.4 ± 0.1
TV23	39.1 ± 1.0	4.9 ± 0.3	19.8 ± 0.9	116.1 ± 0.5	41.1 ± 0.8	220.6 ± 0.6	60.0 ± 1.1	155.1 ± 0.2	0.4 ± 0.1
TV25	52.3 ± 0.6	8.1 ± 0.5	17.0 ± 0.5	122.0 ± 0.3	44.2 ± 0.1	243.7 ± 0.3	61.2 ± 0.2	174.4 ± 0.1	0.4 ± 0.0
TV26	32.1 ± 1.3	3.6 ± 0.6	12.3 ± 0.8	120.1 ± 0.5	41.7 ± 0.2	209.8 ± 0.4	54.0 ± 0.3	152.2 ± 0.4	0.4 ± 0.0
TV30	30.2 ± 1.7	5.1 ± 0.6	12.6 ± 0.8	91.4 ± 1.4	31.9 ± 0.7	175.0 ± 1.5	44.5 ± 0.8	125.4 ± 1.7	0.4 ± 0.1
Ex Cambod^#^	38.9 ± 2.7	1.3 ± 0.2	17.5 ± 1.1	109.0 ± 0.6	28.2 ± 1.0	204.8 ± 1.5	45.6 ± 1.1	157.9 ± 1.8	0.3 ± 0.1
*China varieties *									
TV7	18.2 ± 0.4	8.2 ± 0.7	17.2 ± 0.9	76.7 ± 1.3	45.5 ± 0.8	168.1 ± 1.5	60.7 ± 0.6	98.2 ± 1.8	0.7 ± 0.1
14/13/3	34.7 ± 0.4	2.4 ± 0.7	15.1 ± 1.4	85.0 ± 0.7	24.7 ± 0.6	157.4 ± 0.9	39.8 ± 1.2	119.7 ± 0.6	0.3 ± 0.1
14/100/10	16.5 ± 0.3	3.6 ± 0.7	6.6 ± 0.2	90.0 ± 0.3	25.4 ± 0.5	142.1 ± 0.2	32.0 ± 0.5	106.5 ± 0.4	0.3 ± 0.1
14/100/16	23.4 ± 0.4	2.9 ± 0.1	6.9 ± 0.1	77.6 ± 0.3	31.6 ± 0.4	142.4 ± 0.6	38.5 ± 0.4	101.1 ± 0.5	0.4 ± 0.0
14/100/6	24.3 ± 0.2	5.8 ± 0.1	7.0 ± 0.1	85.8 ± 0.3	25.9 ± 0.2	148.7 ± 0.2	32.8 ± 0.2	110.1 ± 0.3	0.3 ± 0.1
317/7	25.7 ± 0.2	6.1 ± 0.1	7.9 ± 0.3	77.7 ± 0.2	24.4 ± 0.2	141.7 ± 0.1	32.2 ± 0.2	103.5 ± 0.2	0.3 ± 0.0
317/2	31.2 ± 0.1	3.9 ± 0.1	15.4 ± 0.1	89.0 ± 0.1	27.1 ± 0.1	166.7 ± 0.1	42.5 ± 0.1	120.2 ± 0.1	0.4 ± 0.0
317/3	31.2 ± 0.2	2.0 ± 0.3	14.2 ± 0.3	86.5 ± 0.1	20.2 ± 0.1	154.1 ± 0.2	34.3 ± 0.3	117.7 ± 0.1	0.3 ± 0.0
317/4	33.4 ± 0.1	2.1 ± 0.1	7.5 ± 0.3	85.9 ± 0.1	25.2 ± 0.2	154.1 ± 0.2	32.67 ± 0.2	119.3 ± 0.1	0.3 ± 0.0
P126	34.0 ± 0.2	1.8 ± 0.2	15.4 ± 0.2	81.3 ± 0.1	21.2 ± 0.0	153.3 ± 0.1	36.5 ± 0.1	115.4 ± 0.1	0.3 ± 0.0
Vimtal	30.2 ± 0.2	3.0 ± 0.1	15.4 ± 0.1	88.0 ± 0.1	26.8 ± 0.1	157.6 ± 1.1	42.1 ± 0.1	118.6 ± 0.2	0.4 ± 0.0

*CATRAT = (EC+ECG)/(EGC+EGCG); ^#^Ex Cambod: Extreme Cambod.
